# Predictors of Online Cancer Prevention Information Seeking Among Patients and Caregivers Across the Digital Divide: A Cross-Sectional, Correlational Study

**DOI:** 10.2196/cancer.5108

**Published:** 2016-03-09

**Authors:** Tamar Ginossar

**Affiliations:** ^1^ University of New Mexico Cancer Center Department of Communication and Journalism University of New Mexico albuquerque, NM United States

**Keywords:** digital divide, Internet, information seeking behavior, minority health

## Abstract

**Background:**

The digital divide is a recognized public health problem caused by social determinants that exacerbate health disparities. Despite the “tectonic shift” in how most of the public obtains cancer information, underserved communities are at increased risk of being digitally marginalized. However, research that examines factors underlying eHealth information seeking in diverse health contexts is lacking.

**Objective:**

The aim of this paper is to explore preferences and use of eHealth cancer prevention information (CPI) among patients and caregivers attending a minority-serving oncology clinic using the comprehensive model of information seeking as a theoretical framework. Specifically, the study examined the role of social determinants and prevention orientation in differences in preference and use of the Internet for CPI seeking among this diverse sample.

**Methods:**

Survey methodology was used to identify social determinants and behavioral factors, including prevention orientation as correlates and predictors of respondents’ (n=252) preferences and use of eHealth for CPI seeking.

**Results:**

Less than half (112/252, 44.4%) of respondents said that if faced with the need to seek CPI, they would seek this information online. In the final logistic regression model, education, ethnicity, age, and prevention orientation made significant contributions to the model (*P*<.05). Specifically, for each year increase in age, participants were 3% less likely to use the Internet for CPI seeking (*P*=.011). Compared to college graduates, respondents who did not complete high school were 11.75 times less likely to cite the Internet as a CPI carrier (*P*<.001) and those with a high school education were 3 times (2.99, *P*=.015) less likely. In addition, the odds that a Spanish speaker would cite the Internet as a CPI carrier were one-fifth (22%) of non-Hispanic whites (*P*=.032) and about one-quarter (26%) of English-speaking Latinos (*P*=.036). Finally, with each one point increase on the prevention orientation scale, respondents were 1.83 times less likely to cite online CPI seeking (*P*=.05).

**Conclusions:**

Social determinants to health have profound influence on eHealth CPI seeking. Providers and policy makers should focus on meeting patients and family members’ CPI needs following diagnosis and increase eHealth accessibility and availability of evidence-based CPI to diverse populations. Future research is needed to unravel further differences in eHealth CPI seeking, including those among Native Americans that emerged as an additional digitally underserved racial/ethnic group. Finally, additional factors underlying these differences should be explored to better tailor CPI eHealth information to diverse communities’ information needs.

## Introduction

### Overview

The exclusion of ethnic and racial minorities and vulnerable populations from accessing Web-based health information [1-3] exacerbates cancer-related health disparities. Despite a “tectonic shift” in how cancer patients and their families obtain information and make decisions about their health, including primary and secondary prevention [4], 1 in 5 American adults does not use the Internet. These nonusers are disproportionally likely to be senior citizens, Spanish speakers, adults with less than a high school education, people with low income [5], and cancer survivors [6]. In view of the mounting evidence on the prevalence and demographic predictors of online health information inequities in the general public [3], scholars noted the importance of examining the digital divide in communities that cope with specific health concerns [7].

The goal of this study is to examine use of the Internet for cancer prevention information (CPI) seeking among cancer patients and their families attending a minority-serving academic cancer center in New Mexico, United States, a minority-majority state with the lowest national rate of Internet access at home [8]. The need for this study stems from the importance of eHealth CPI for people diagnosed with cancer and their families. CPI is necessary to inform lifestyle- and screening-related behavior changes. Adhering to evidence-based preventive practices may reduce the likelihood of cancer reoccurrence following remission, reduce second primary cancer diagnosis among cancer survivors, improve overall health outcomes, and reduce anxiety among this population [9,10]. Although people diagnosed with cancer and their families consistently report that they are interested in CPI [11], these needs are rarely met in the medical encounter [12]. A recent study revealed that ethnic and racial minority patients and caregivers value CPI but are often blocked from seeking it [13]. It is likely that the digital divide contributes to the barriers they experience, but past studies did not explore use of the Internet for CPI seeking in this population.

Consistent with the theoretical framework of the comprehensive model of information seeking (CMIS) [14,15], this study aimed at understanding preferences and actual use of the Internet for CPI seeking among diverse patients and their families with the goal of informing a future intervention. Following the CMIS, this study examined factors that are consistent with social determinants to health and have been shown to distinguish between eHealth users and nonusers [16] as well as additional behavioral- and individual-level factors including past CPI-seeking behaviors and prevention orientation [17-19].

### Theoretical Background

The CMIS [14,15] provides a theoretical framework for this research developed to explain and predict cancer-related health information seeking and source utilization and has been tested in diverse settings [14,15,20-23] including online communication environments [24]. Integrating concepts from uses and gratification research, the health belief model, and a model of media exposure and appraisal, the CMIS proposes three primary levels of variables that influence cancer information seeking. Antecedent factors, such as demographics, beliefs, and attitudes constitute the first level, factors related to the information sources comprised the second level, and the third level includes specific information-seeking behaviors [15,25]. According to this model, the antecedents determine the underlying imperatives to seek information; the perceived characteristics of the information carriers influence the intentions to seek information from particular carriers; and the information-seeking actions are the outcomes of the antecedents and the characteristics of the information carriers [21].

### Cancer Prevention Information Seeking Among Survivors and Caregivers

Whereas some patients might perceive CPI as irrelevant or stressful [26,27], review of the literature concluded that CPI constitutes an important information need, comprising 30% of patient information needs [11]. This information need is likely related to the increased risk of secondary diagnoses and reoccurrence following remission among cancer survivors [11]. Additionally, people diagnosed with cancer might also be interested in receiving information in order to support their families in preventing cancer or to better understand the etiology of their illness. Diagnosis of cancer in the family also leads to heightened perceptions of cancer risk and cancer worry among family members [28], which often result in CPI needs [29]. Obtaining CPI is necessary in making informed decisions about cancer prevention and can reduce anxiety [9], but only one-fifth of oncologists provide this information [12].

Cancer patients vary their source utilization in accordance with the type of information they seek [30]. It is therefore essential to examine use of the Internet for CPI seeking, but most studies to date focused on general cancer information seeking among cancer patients [6,22,31-33]. Additionally, studies that examined CPI seeking and source utilization following diagnosis recruited predominantly non-Hispanic white college graduates [22,30,34,35], and their findings might not be generalized to more diverse patient populations. A recent community-based study of CPI seeking among a diverse sample of cancer patients and their families documented that ethnic minorities and those with lower social economic status (SES) were less likely to seek CPI compared to other respondents despite equal or higher perceptions of its importance and motivation to receive it [13]. Experiencing barriers to online access might contribute to this disparity [6], but eHealth preferences and use of CPI seeking following diagnosis among diverse people diagnosed with cancer and their loved ones was not previously examined. According to the CMIS [14,15], information seeking along the cancer continuum involves different mindsets and is motivated by different needs. Therefore, it is important to better understand CPI seeking following diagnosis with cancer rather than assume it follows patterns identified in different populations or cancer information seeking contexts. Such an understanding is essential for designing health communication and education efforts for this population.

According to the CMIS, there are inherent differences in cancer information seeking among the general public and those who cope with a cancer diagnosis. Whereas the first type of information seeking is taking place “when someone is not confronted with the symptoms or disease, but may be mildly concerned with prevention [21],” persons who seek information following diagnosis cope with a pressing and acute problem, which is “novel and fraught with emotional complications [21].” In view of this difference in information seeking that distinguishes those who seek CPI following cancer diagnosis from the general public, it is important to explore CPI seeking following cancer diagnosis. Use of the CMIS to examine use of the Internet for CPI seeking can shed more light on this important experience and its predictors.

### Antecedents to Internet Use in Cancer Prevention Information Seeking

Consistent with the CMIS, this study examines whether certain antecedents are related to seeking CPI online among cancer patients and their families who attend an ambulatory care oncology clinic at a National Cancer Institute (NCI)-designated minority-serving cancer center. The following factors were identified in past research that identified social determinants of CPI seeking online among the general population [36-39] and among those following diagnosis [40].

#### Social Determinants of Online Cancer Prevention Information Seeking

As previously mentioned, older adults, those with low SES or health status, and cancer survivors are less likely to seek health information online [25,41,42]. Ethnic minority cancer patients and their families have lower rates of accessing the Internet to seek cancer-related information compared to non-Hispanic whites [43]. According to national survey data, Latinos are considered the most digitally disadvantaged ethnic group in the United States [44]. In addition to having less access to the Internet, Latinos also hold different perceptions of its utility [45]. These disparities are related to inequalities in the propensity to seek CPI and lack of availability of Spanish language information resources [39]. Because English emerges as the most significant predictor of health information seeking among Latinos [46,47], it is important not to conflate Latino ethnicity with limited English proficiency and to examine experiences of monolingual Latino Spanish speakers as well as those who are fluent in English. Whereas national surveys document the digital divide among Latinos, telecommunication evidence suggests that Native American communities are the most digitally marginalized [48]. Because they are not included in most national surveys such as those conducted by the NCI [33,49-58] and the Pew Research Center [5,46,59,60], the impact of the digital divide on health information seeking among Native Americans remains largely unknown.

#### Motivation to Seek Cancer Prevention Information

In contrast to the role of social determinants of cancer health information-seeking behavior and the digital divide including race/ethnicity, education, income, and other demographic factors [33], not much is understood about the role of other psychosocial factors in seeking CPI online. A potential antecedent that can relate to CPI seeking online is motivation, an important variable in health information seeking [61]. Individuals’ motivation to be healthy leads to their interest in health issues and active engagement in information seeking, including selection of new information technologies [17]. Specifically, individuals’ prevention orientation, or health consciousness, is likely to influence online CPI seeking [62]. A construct of health orientation [17], prevention orientation measures the degree to which individuals feel that preventive behavior is important to them and worthy of engaging in. It is thought to be a personality construct that is influenced by specific health contexts [17]. Prevention orientation is related to different health behaviors including online support group participation [17,62] and CPI seeking among minority cancer patients and caregivers [13]. Research revealed that among the general population, health information seeking online is related to higher levels of health orientation [63]. However, it is unknown to what degree it is related to use of the Internet as a CPI carrier.

Following the CMIS [14,15] and previous studies that examined the manifestation of digital inequities in cancer information seeking as described above [22,25,64], the current research goal was to examine predictors of online CPI seeking among a diverse sample of people diagnosed with cancer and their families. Moreover, research indicated the importance of understanding individuals’ preferred sources, conceptualized as information sources they indicate they would use if faced by an information need as well as the information sources they actually use [33]. Therefore, this study aimed at understanding both correlates and predictors of preference for the Internet as a CPI carrier and differences among those who sought CPI online and those who sought it offline. To learn about eHealth CPI seeking among a diverse sample of people diagnosed with cancer and their families, the following research questions were posed:

RQ1: What are the factors associated with citing the Internet as a CPI carrier among cancer patients and their families?

RQ2: What are the factors associated with online and offline CPI seeking among cancer patients and their families?

## Methods

### Overview

This is a cross-sectional, correlational study using secondary data analysis of a study that examined CPI seeking among a diverse sample of people diagnosed with cancer and their families. Individuals were eligible to participate if they were 18 years of age or older and receiving care at a NCI-designated minority-serving cancer center in New Mexico (referred to as patients) or were accompanying a person receiving care (referred to as caregivers). The study was approved by the institutional review boards of the University of New Mexico and its cancer center, and informed consent was obtained from all participants.

### Recruitment

Direct recruitment approach [65,66] and the screening strategy for oversampling minority participants [67] were used to recruit hard-to-reach patient populations [65,66] in the context of ethnic-related health disparities [68-70]. Response rate was 91%. Patients and caregivers who declined cited reasons including lack of English/Spanish proficiency, emotional state, and lack of interest. Of the 252 individuals recruited, 105 were caregivers. The largest group (56/105, 53.3%) included spouses of patients, followed by adult children (29/105, 27.6%) and other family members (20/105, 19.0%). One caregiver identified as a friend.

Participation included answering a survey in English (214/252, 84.9%) or in Spanish (38/252, 15.1%), according to participants’ preference. A bilingual, bicultural research team member was available to answer questions and requests for clarification. Participants were offered to have the survey read by the interviewer/research team member. A total of 54 participants preferred to have the survey read to them, and a team member read each question out loud, ensured that participants understood the questions, and filled in the participants’ answers. Respondents received a $10 gift card to a local grocery store as compensation for participation. Participants’ demographic information is presented in Table 1.

**Table 1:** Personal characteristics.

		N (%)
**Gender**		
	Women	94 (37.3)
	Men	157 (62.3)
**Patient/relative**		
	Patient	144 (57.1)
	Relative	108 (42.9)
**Marital status**		
	Married/live with a partner	145 (57.5)
	Not married	100 (39.7)
	Missing	7 (2.8)
**Race/ethnicity**		
	American Indian/Native American	17 (6.7)
	Latino-Spanish speaking	36 (14.3)
	Latino-English speaking	131 (52.0)
	Other (African American & Asian)	10 (4.0)
	Non-Hispanic white	58 (23.0)
**Education**		
	Less than high school	50 (19.8)
	High school graduate	59 (23.4)
	Some college/training	83 (32.9)
	College graduate	55 (21.8)
	Missing	5 (2.0)
**Annual household income**		
	<$20,000	115 (45.6)
	$20,001-$35,000	39 (15.5)
	$50,001-$70,000	28 (11.1)
	>$70,001	29 (11.5)
	Missing	3 (1.2)
**Medical insurance**		
	Uninsured	48 (19.0)
	Insured	202 (80.2)
	Missing	
**Language of survey**		
	English	214 (84.9)
	Spanish	38 (15.1)

### Measures

#### Antecedents

Sociodemographic questions pertained to gender, age, education, household income, marital status, and clinical information including self-reported health status. The following measures were used to examine perceptions and experiences that might influence CPI seeking online.

Behavioral-related measures consisted of prevention orientation, previous CPI seeking, past use of the Internet for CPI seeking, and health information-seeking experiences. All the survey measures have been tested in prior research and were reliable and valid, as described in the following sections.

Prevention orientation related to respondents’ motivation to engage in healthy behaviors including health information seeking. It was measured by the prevention orientation subscale, which was previously tested and validated [17] and comprises five items. Examples include “Living life in the best possible health is very important to me” and “Eating right, exercising, and taking preventive measures will keep me healthy for life.” Responses were measured on a scale ranging from 1 to 5 with 1 representing *strongly disagree* and 5 representing *strongly agree*. The Cronbach alpha for this measure was .88.

Past CPI-seeking behavior was measured using Health Information Trends Survey (HINTS) items [33,52]. Participants were asked whether they have looked for information about cancer prevention: “Have you ever looked for information about cancer prevention?” and ”When was the last time you searched for information about how to prevent cancer?” Responses to the second question were coded as *prior CPI seeking* when respondents indicated a time when they sought CPI and *no prior CPI seeking* when they did not indicate such search. Cronbach alpha was assessed at .98.

Health information-seeking experiences were measured by examining respondents’ evaluation of the difficulty of CPI seeking. The 6 HINTS items included statements such as "It took a lot of effort to get the information that I needed" and “The information I found was too hard to understand.” This scale reliability has been previously tested [71] and determined to have a Cronbach alpha of .84.

#### Outcome Measures

Selection of the Internet as a CPI carrier was the primary outcome measure. Following the HINTS instrument that examined cancer information seeking [72], we asked all respondents where they would go first for information about cancer prevention and asked those who previously sought CPI to indicate the information sources they used [72]. Responses to these open-ended questions were coded as a binary variable that referred to whether respondents listed the Internet as a CPI source or not. The coders were two doctoral research assistants, and they met for one training session that consisted of directions on coding the responses as *online* when participants’ referenced the Internet. Such references included listing specific websites or mentioning the Internet as a general information source for CPI. Coder were instructed to code entries as *offline* when no reference to online sources was mentioned. Following this session, they coded the responses individually and met again to compare their results. Intercoder reliability was computed at 98%; the Krippendorff alpha was .96 **.**

### Analysis

Analysis was conducted using SPSS version 22 software (IBM Corp). First, to determine the sample’s demographic characteristics, chi-square tests were used to examine categorical variables and *t* tests were used to examine continuous variables. The main outcome of the analysis of the first research question was whether respondents cited the Internet as a CPI carrier they would turn to if faced with a CPI need. These results are listed in Multimedia Appendix 1.

Missing values ranged from 0% to 5% in all variables, so listwise deletion was used for categorical variables and mean substitution was used for continuous variables. The final logistic model indicates those variables remaining statistically significant at the .05 level and is presented in Multimedia Appendix 1.

The main outcome in answering the second research question included actual use of the Internet by respondents who sought CPI. Analysis of differences between online and offline CPI seekers was conducted using the *t* test and chi-square test as indicated.

## Results

### Selecting the Internet As a Cancer Prevention Information Carrier

To answer the first research question, which examined factors that distinguished respondents who cited the Internet as an information carrier they would turn to if faced with a CPI need from those who did not, analysis of the answers by all respondents to the survey (n=252) was performed. Less than half (112/252, 44.4%) of respondents said that if faced with the need to seek CPI, they would seek this information online. Differences between respondents who cited the Internet as a CPI carrier and those who did not are described in the subsequent section and detailed in Multimedia Appendix 1.

### Demographic Variables

Women (74/157, 47.0%) and men (38/93, 41.0%) did not differ significantly in citing the Internet as a CPI source, as indicated in the chi-square test (χ^2^_1,N=251_=0.93, *P=*.36)*.* Family members (56/108, 52.0%) were more likely than patients (44/143, 39.0%) to report that they would use the Internet for CPI, with a chi-square test showing that this difference was statistically significant (χ^2^_1,N=251_=4.01, *P<*.05)*.* Differences in marital status of respondents who cited the Internet as a CPI source (71/147, 48.1%) and those who did not (39/100, 39.0%) were not statistically significant as indicated in chi-square analysis (χ^2^_1,N=247_=2.08, *P*=.15)*.*

Analysis of ethnic differences in citing the Internet as a CPI source revealed that non-Latino whites (35/78, 60%), followed by Latinos who answered the survey in English (64/128, 50.0%) were the most likely to select the Internet as a CPI source, compared to about one-third of Native Americans (6/17) and African Americans and Asians (3/10) and only 10% of Latinos who filled in the survey in Spanish (4/38). Chi-square analysis revealed that ethnic differences in selecting Internet for CPI seeking were statistically significant (χ^2^_4,N=251_=26.64, *P=*.00).

Level of education attained was related to respondents’ indication that they would use online CPI sources. Whereas only one-tenth (5/52) of those who did not graduate high school cited the Internet as a CPI carrier they would turn to, almost half (26/59, 44%) of high school graduates, over half (43/83, 52%) of those with some post high school education, and two-thirds (37/56, 67%) of college graduate respondents would use online CPI sources. Chi-square analysis revealed that differences in educational attainment between those selecting Internet for future CPI seeking and those who did not were statistically significant (χ^2^_3,N=249_=38.96, *P=*.00)*.*

Income was also related to citing the Internet as a CPI source. Less than one-third (34/117) of those who have annual household income of less than $20,000 said they would use the Internet for CPI seeking, but 71% of those in the highest income bracket in this study would seek CPI on the Internet (27/38). Additional differences are presented in Multimedia Appendix 1.

Differences in income levels in citing the Internet as a CPI source were statistically significant (χ^2^_4,N=251_=28.26, *P*=.00). Respondents with medical insurance were more likely to cite the Internet as a CPI carrier (47.5%) than the uninsured (29.2%). Chi-square testing showed that this difference was statistically significant (χ^2^_1,N=250_=5.30, *P*<.05)*.* Almost half of insured respondents (96/199) cited the Internet as a CPI source compared to 29% of uninsured respondents (14/48). Chi-square test revealed that this difference was statistically significant (χ^2^_1,N=250_=5.30, *P*<.05)*.*

Language of survey was significantly associated with citing the Internet as a CPI carrier. Half of participants (108/213) who answered the survey in English cited the Internet as a CPI carrier they would turn to compared to only a tenth of respondents who answered in Spanish (4/38). The chi-square test showed that the difference was significant (χ^2^_1,N=247_=5.70, *P*<.05)*.*

Participants who cited the Internet as a CPI source were significantly younger (mean 51.2 years [SD 12.56]) than those who would not use the Internet for CPI seeking (mean 55.8 [SD 12.99], *t*_249_=−2.82, *P*<.01). Time since diagnosis of those who cited the Internet as a CPI sources (mean 32.09 months [SD 4.24]) and of those who did not cite the Internet (mean 29.40 [SD 38.45]) was not statistically significant (*t*_241_=0.52, *P*=.60).

Individuals who cited the Internet as a CPI source had better self-reported health (mean 2.73 [SD 1.00]), compared to those who did not think they would use the Internet as a CPI source (mean 3.18 [SD 1.01]). A *t* test revealed that these differences were statistically significant (*t*_248_=−3.52, *P*<.01). Individuals who cited the Internet as a CPI carrier had lower levels of prevention orientation (mean 4.17 [SD 0.54]) compared to those who would not search CPI online (mean 4.34). These differences were statistically significant (*t*_247_=−3.41, *P*<.01).

Almost half of participants who sought CPI in the past believed that they would use the Internet for CPI seeking if faced with CPI need compared to 41% of those who did not previously sought CPI (n=46/113), a difference that did not reach statistical significance as indicated in chi square analysis (χ^2^_1,N=249_=1.48, *P*=.25).

Participants who used the Internet in the past for CPI seeking were more likely to cite the Internet as a CPI carrier compared to those who did not previously use the Internet for CPI seeking. Only 28% (18/58) of those who did not use the Internet in the past to seek CPI believed that they would use it in the future, whereas a majority (46/64, 72%) of those who sought CPI online in the past responded that they would use it in the future. Chi-square analysis revealed that these differences were statistically significant (χ^2^_(1,N=246)_=16.55, *P*=.00).

### Predictors of Selecting the Internet for Cancer Prevention Information Seeking

Logistic regression analysis was conducted to answer the first research question and determine which independent variables predicted citing the Internet as a CPI carrier. Included were variables significantly related to differences in citing the Internet as a CPI carrier in the previous analysis (status as patient or caregiver, race/ethnicity, education, age, health status, and prevention orientation). Income and medical insurance were not included due to multicollinearity. A test of the full model against a constant only model was statistically significant, indicating that the predictors as a set reliably distinguished between participants who cited the Internet as a CPI carrier they would turn to and those who did not (χ^2^_8,N=247_=71.7, *P*=.00, *Nagelkerke* R^2^=.319).

Education, ethnicity, age, and prevention orientation made significant contributions to the model (*P*<.05). Specifically, the odds of citing the Internet as a CPI carrier are lower with age. For each year increase in age, participants are 3% less likely to use the Internet for CPI seeking (*P*=.011). Education also contributed significantly to the model. Compared to college graduates, respondents who did not complete high school were 11.75 times less likely to cite the Internet as a CPI carrier (*P*=.00), and those with a high school education were three times (2.99, *P*=.015) less likely to cite the Internet as a CPI carrier compared to college graduates. The odds that a Spanish speaker would cite the Internet as a CPI carrier were one-fifth (22%) of that of non-Hispanic whites (*P*=.032) and about one-quarter (26%) of English-speaking Latinos (*P*=.036). Other ethnic/racial differences were not significant. Additionally, with each one point increase on the prevention orientation scale, respondents were 1.83 times less likely to cite online CPI seeking (*P*=.05). Other variables did not contribute to prediction of CPI in a statistically significant way. The results are presented in Multimedia Appendix 2.

### Actual Use of the Internet for Cancer Prevention Information Seeking

Of the 252 respondents to the survey, more than half (133, 52.8%) indicated they have previously sought CPI. These individuals were asked where they sought CPI, and their responses were analyzed to understand the characteristics of online CPI seekers, defined as those who used the Internet to seek CPI, versus offline CPI seekers, those who used only non-Internet CPI sources. These differences are reported in Multimedia Appendix 1. As indicated in the appendix, most CPI seekers (75/133, 56.3%) used the Internet to search for CPI.

The majority of women who sought CPI used the Internet (55/85, 65%) in contrast to a minority of men (20/48, 42%). These differences were statistically significant as indicated in chi-square analysis (χ^2^_1,N=133_=6.62, *P*<.05). However, family members who sought CPI were not significantly more likely to use the Internet (37/61, 61%) compared to patients (38/72, 53%) (χ^2^_1,N=133_=0.83, *P*=.38). Similarly, marital status was not related to CPI seeking online in a statistically significant way (χ^2^_1,N=133_=1.08, *P*=.37).

Examination of the relationship between ethnic identity and online CPI seeking indicated that non-Latino white CPI seekers were most likely to use the Internet (26/39, 67%), followed by Latinos who completed the survey in English (39/56, 59%). In comparison, a minority of Latinos who responded in Spanish (5/13, 39%), Native Americans (3/9, 33%) and African Americans/Asians (2/6, 33%) who sought CPI did so online. Chi-square analysis revealed that ethnic differences among online and offline CPI seekers did not reach statistical significance (χ^2^_4,N=133_=6.81, *P=*.146).

Online information seeking was positively associated with educational attainment. Among online CPI seekers, only one had less than high school education (7%). A minority of high school graduates sought CPI online (9/23, 39%). In contrast, a majority of participants with some post high school training or education (37/57, 65%) and of those who were college graduates (38/56, 71%) were online seekers. Chi-square analysis indicated that these differences were statistically significant (χ^2^_3,N=132_=21.56, *P*=.00).

Online information seeking was positively associated with annual household income. A minority (21/54, 39%) of CPI seekers with an annual household income less than $20,000 used the Internet for CPI seeking compared to a vast majority (23/27, 85%) of CPI seekers with an annual household income above $70,000. Chi-square analysis revealed that these differences were statistically significant (χ^2^_4,N=132_=16.05, *P<*.01). This information and information about additional income brackets is presented in Multimedia Appendix 1. In addition, medically insured and uninsured CPI seekers were equally likely to seek CPI online, with 56.5% of CPI seekers using the Internet regardless of their medical insurance status (χ^2^_1,N=133_=.00, *P*=1.00).

Although the majority of CPI seekers who answered the survey in English were online CPI seekers (70/120, 58.3%) compared to a minority of respondents who filled in the survey in Spanish (5/13, 39%), the results did not reach statistical significance (χ^2^_1,N=133_=1.88, *P*=.24). Online information seekers were younger (mean 52.7 [SD 12.30]) than offline CPI seekers (mean 56 [SD 11.90]), but a *t* test indicated that these results did not reach statistical significance (*t*_133_=−1.55, *P*=.123). Time since diagnosis was longer (mean 32.9 [SD 38.22]) among online CPI seekers compared to offline CPI seekers (mean 26.72 [SD 35.22]). These results were not statistically significant (*t*_133_=0.948, *P=*.345). An additional factor that differed between online CPI seekers and offline CPI seekers was health status, with online information seekers reporting better health (mean 2.6 [SD 0.94]) compared to offline CPI seekers (mean 3.2 [SD 0.93]). This difference was statistically significant (*t*_133_=3.52, *P*<.01).

The analysis revealed that offline CPI seekers had higher levels of prevention orientation (mean 4.42 [SD 0.44]) compared to online CPI seekers (mean 4.27 [SD 0.50]), and this difference was statistically significant (*t*_130_=−1.80, *P*<.05). Finally, offline and online CPI seekers reported the same levels of difficulty in accessing information (mean 2.8 [SD 0.94 and 0.82, respectively], *t*_130_=−0.04, *P*=.97). The results are presented in Multimedia Appendix 1.

## Discussion

### Principal Findings

The current study applied the CMIS to expand the knowledge on the digital divide in specific clinical settings [25] by examining eHealth use for CPI seeking among an ethnically and socioeconomically diverse sample of people diagnosed with cancer and their caregivers who attend a minority-serving academic cancer center in New Mexico. Borrowing from the CMIS framework, this study examined factors related to social determinants of health and motivation as potential antecedents to CPI seeking. According to the CMIS, certain antecedents determine the type of information sources and channels used as well as overall information behavior. The findings revealed the importance of social determinants as antecedents to CPI eHealth seeking in this population. Consistent with patterns identified in past research on health information behavior and the digital divide [22,25], certain demographic factors, including race/ethnicity, educational attainment, household income, age, gender, and health status are related to the propensity to seek cancer information online. In the regression model, younger age, higher levels of education, being non-Hispanic white or English-speaking Hispanic compared to Spanish-speaking Latino, and reporting better health status were significant predictors of CPI eHealth seeking. These factors have been shown to predict disparities following cancer diagnosis in other cancer-related topics [1,6]. Therefore, these findings demonstrate how disparities in use of the Internet are merely one factor within an overall inequity in health information seeking and contribute to the literature that documents how the “double divide” is blocking those who need this information the most from accessing it.

Further, this study contributes to past research that suggested that the CMIS is a useful framework for understanding cancer patients’ information source selection and usage on the cancer continuum [24]. This model was originally applied to selection of mass media sources [14], but a growing body of research applies this framework to understand use of online cancer information sources [24,31]. The current study reveals the importance and utility of integrating factors related to social determinants to the CMIS in exploring cancer information behavior of diverse populations. An additional strength of the CMIS is its structural flexibility. Applying the CMIS in diverse contexts allows for examination of specific predictors of information behavior. The model highlights the importance of incorporating a variety of factors to account for information seeking across different contexts and the fact that the specific patterns of relationships among variables in the model are contingent on the context [21]. Therefore, the current findings point at the importance of examining CPI seeking of underserved cancer patients and their families rather than assume that their information seeking parallels those of the general public. For instance, in contrast to studies utilizing CMIS that reported no significant correlation between antecedents and characteristics of cancer information sources among the general public [14], this study documented the importance of social determinants in predicting online CPI.

Applying the CMIS framework also led to a deeper understanding of the differences between actual CPI versus hypothetical selection of sources. Clearly, not all individuals are able to seek information or to enact on their preferred information sources. Past research documented some discrepancies between cancer information sources that individuals indicate they would select for actual use [73]. This study examined information preferences along with self-reports of actual use. Participants’ perceptions of whether they would use the Internet for CPI seeking were largely consistent with reports of use among CPI seekers, with some exceptions. Ethnicity, income, education, and health status were related both to the rates of citing the Internet as a potential CPI source and reports of its use among CPI seekers. However, some differences were noted. First, although men and women did not differ in a statistically significant way in citing the Internet as a CPI source they would use, women who sought CPI were significantly more likely to use the Internet than men who sought CPI. In addition, although having medical insurance was related to citing eHealth as a CPI source, online and offline CPI seekers were equally likely to have medical insurance. Similarly, family members were more likely to cite the Internet as a CPI source, but family members did not differ significantly from patients choosing to be online versus offline CPI seekers. These findings point at the importance of examining information source preferences as well as actual use.

Exploring different psychosocial factors as predictors of information seeking is an important part of studies utilizing the CMIS [14,15] as well as other research of cancer information behavior. An intriguing finding in the current study relates to the negative correlation between prevention orientation and online CPI seeking. In other words, participants who indicated use of the Internet for CPI seeking scored lower on prevention orientation than those who did not report Internet use. This finding conflicts with past studies that indicated the importance of psychosocial factors on health information behavior following cancer diagnosis [34]. Prevention orientation was positively associated in past studies with seeking CPI even after controlling for other factors [13]. This finding further underscores the importance of considering both context and specific predictors because certain psychosocial factors might be associated differently with certain cancer information behaviors based on the context and content of the information and the information sources utilized. Future studies and in particular studies using a long attitudinal design should examine whether online CPI seeking increases skepticism regarding the importance of prevention and how it might influence cancer prevention behavior such as diet, exercise, and screening practices.

These findings also contribute new insights into the dynamics of the digital divide. Age was a key factor that discriminated between those citing the Internet as a CPI source and those who did not in both bi-variate and multivariate analyses. However, this difference is smaller than previously reported. Specifically, persons who cited the Internet as CPI carrier were on average only 4 years younger than those who did not compared to 10 years’ difference reported in past studies [25]. Moreover, difference in age among online and offline CPI seekers was smaller and did not reach statistical significance. This finding is consistent with the prediction that age differences in Internet use would decline over time as older adults increasingly use the Internet [25]. The findings indicate a trend toward persistence of the digital divide in CPI seeking because respondents who previously sought CPI online were more likely to indicate that they would seek CPI online in the future. Future studies should explore the role of intervention in decreasing the digital divide and the impact of such intervention on CPI behavior and prevention-related behaviors such as participation in screening and lifestyle changes.

The results also revealed better self-reported health status among those who cited eHealth as a CPI source versus those who did not. Previous research reported conflicting findings regarding the relationship between health status and online health information seeking [74,75]. It is possible that lower health status is related to seeking treatment information online whereas those who feel healthier focus more resources on CPI seeking. Alternatively, it is possible that those with better health have better access to the Internet or that respondents who seek CPI online are better able to maintain their health. This relationship should be explored in future studies using larger cohorts and following them over time.

### Practical Implications

These disparities in CPI seeking online are particularly concerning because comprehensive cancer centers charged with targeting communities with prevention initiatives do not have overarching strategies to disseminate CPI to cancer survivors and their families [76]. Researchers reported that when barriers to access are removed, the benefits of online information seeking are extended to individuals from underserved communities [24]. Therefore, programs should be designed that consider CPI sources available to the target audiences. While many patients and family members turn to the Internet to seek this information, those who are disfranchised and need this information the most are least likely to receive it.

Since interventions are likely to be conducted in specific geographic communities [77], it is essential that research incorporates understanding of CPI seeking among clinical populations in certain locales that necessitate effective recruitment strategies. In contrast to past research that used nationally or state representative phone-based [25,33,49,51,52,54,55,57,58] or mailed surveys [30,34,58], this study employed direct recruitment methods in a specific geographic community [65,66], an approach that has been shown to include individuals from underserved communities [70,78]. This approach facilitated a high response rate among this hard-to-reach population of patients and caregivers. Future interventions should apply similar recruitment methods and measure their effectiveness in engaging individuals.

### Limitations and Future Research

Due to this study’s focus on a specific geographical region, its sample consisted entirely of patients and caregivers who attend a minority-serving cancer center in New Mexico. Whereas this focus enriches understanding of specific communities and allows for design of community-based interventions, this sample may not be representative of cancer patients from other regions along certain dimensions, such as the health and digital disparities they experience. Despite sample size that exceeded the initial power analysis to detect differences in this sample, the large proportion of ethnic minority respondents who did not seek CPI likely rendered certain differences between online and offline CPI seekers statistically insignificant. This limitation is concerning as Native Americans are underserved and underresearched in both digital access and health information research. The current findings point to the disparities experienced by Native American respondents, and more research is needed to provide insight on actual use of eHealth for CPI seeking among Native American cancer patients and their families.

Further, this study relied exclusively on self-reported data, which have documented shortcomings including recall bias and social desirability influences. Triangulation of data revealed consistency in participants’ accounts of their experiences in information seeking, but ultimately the study reports on respondents’ perceptions only. Future research should examine the association between CPI-seeking behavior and additional factors that were not explored in this or in previous studies. For instance, this study did not focus on comparisons between CPI seeking among individuals who cope with different types of cancer, which is associated with differences in seeking general cancer information among patients [34] and might also be related to differences in CPI seeking. In addition, Internet access was not measured in a secondary data analysis; therefore, the reasons behind the reported disparities in eHealth CPI seeking are unknown. Future studies should examine Internet access as well as additional factors that might influence eHealth use including digital and health literacies. This study followed past research that examined disparities in information seeking in binary terms [22,25], a methodological choice that introduced an additional limitation. The digital divide consists of a spectrum of inequalities in use of the Internet [5], and research should examine the nuances of CPI seeking and use of eHealth as well as the relationship between CPI seeking, the specific types of CPI sought, sources used, and outcomes such as knowledge and prevention practices.

## Appendix

Multimedia Appendix 1 Online and offline CPI seeking among cancer patients and relatives.

Multimedia Appendix 2 Logistic regression analysis predicting digital divide in citing the Internet as CPI carrier among cancer patients and relatives.

## Abbreviations

CMIS: comprehensive model of information seeking
CPI: cancer prevention information
HINTS: Health Information Trends Survey
NCI: National Cancer Institute
SES: social economic status
