# Supplementary material for: Predictors of Online Cancer Prevention Information Seeking Among Patients and Caregivers Across the Digital Divide: A Cross-Sectional, Correlational Study
Source: JMIR Cancer. 2016 Mar 9;2(1):e2. doi: 10.2196/cancer.5108 (PMC5369630; doi:10.2196/cancer.5108)
Supplement: Multimedia Appendix 1 [file cancer_v2i1e2_app1.pdf]

Table 2. Online and offline CPI seeking among cancer patients and relatives

	Citing CPI Seeking Sources					Actual CPI Seeking				
	**Online**		**Offline**		**Sig. (p)**	**Online**		**Offline**		**Sig. (p)**
	**N**	**%**	**N**	**%**		**N**	**%**	**N**	**%**	
**Overall**	**112**	**45**	**137**	**55**						
**Gender**					.36					.012
Women	74	47	83	53		30	35	55	65	
Men	38	41	55	60		28	58	20	42	
**Patient/caregiver**					.05					.38
Patient	56	39	87	61		34	47	38	43	
Caregiver	56	52	52	48		24	39	37	61	
**Marital status**					.15					.37
Married/live with a partner	71	48	76	52		31	41	45	51	
Not married	39	39	61	61		27	50	27	50	
**Race/Ethnicity**					.00					.146
Hispanic-English speaking	64	50	64	50		27	41	39	59	
Hispanic-Spanish speaking	4	10	34	90		8	62	5	38	
Native American	6	35	11	65		6	67	3	33	
Non-Hispanic White	35	60	23	40		13	33	26	67	
Other	3	30	7	70		4	67	2	33	
**Education***					.00					.00
Less than high school	5	10	47	90		13	93	1	7	
High school graduate	26	45	33	56		14	61	39	9	
Some college/training	43	52	40	48		20	35	37	65	
College graduate	37	67	18	33		11	29	27	71	
**Annual household income**					.00					.003
<$20,000	34	29	83	71		33	61	21	39	
$20,001-$35,000	17	44	22	56		8	40	12	60	
$50,001-$70,000	19	66	10	34		8	44	10	56	
>$70,001	27	71	11	29		4	15	23	85	
**Medical insurance**					.023					1.00
Insured	96	48	103	52		62	56	48	44	
Uninsured	14	29	34	70		13	56	10	43	
**Language of survey**					.00					.24
English	108	51	105	49		50	42	70	58	
Spanish	4	10	34	90		8	82	5	38	
	**Mean**	**Std.**	**Mean**	**Std.**		**Mean**	**Std.**	**Mean**	**Std.**	
**Age** (Mean in years)	51.24	12.56	55.83	.12.1	.01	52.8	12.64	55.45	12.64	.12
**Months since diagnosis**	32.10	42.45	29.4	38.46	.60	32.94	38.22.	26.72	35.22	.34
**Health Status** (1=excellent)	2.73	.99	3.17	1.01	.001	2.60	.92	3.13	.96	.002
**Previous CPI seeking**					.25	**Not applicable**				
CPI seeker	64	48	68	52						
Non CPI seeker	46	41	67	59						
**Previous online CPI seeking**					.00	**Not applicable**				
Past online CPI seeking	46	72	18	28						
Past offline CPI seeker	18	28	40	59						
**CPI Importance**	4.28	.92	4.50	.711	.03	4.41	.91	4.5	.67	.31
**Prevention orientation**	4.17	.537	4.35	.47	.006	4.25	.50	4.42	.44	.037

**Experiences of CPI seeking (5 = Most difficult)**	**Not applicable**	3.59	.46	3.67	.51	.327
