# Supplementary material for: Predictors of Online Cancer Prevention Information Seeking Among Patients and Caregivers Across the Digital Divide: A Cross-Sectional, Correlational Study
Source: JMIR Cancer. 2016 Mar 9;2(1):e2. doi: 10.2196/cancer.5108 (PMC5369630; doi:10.2196/cancer.5108)
Supplement: Multimedia Appendix 2 [file cancer_v2i1e2_app2.pdf]

Table 3 Logistic regression analysis predicting digital divide in citing the Internet as CPI carrier among cancer patients and relatives

							95% CI. For exp β	
**Variable**	β	**S.E.**	**Wald**	**df**	**Sig.**	**Exp(β)**	**Lower**	**Upper**
Age	.032	.012	6.441	1	.011	1.032	1.007	1.058
General health	.181	.167	1.183	1	.277	1.199	.865	1.662
Patient/relative	.188	.321	.344	1	.557	1.207	.643	2.266
Education			16.713	3	.001			
< high school graduate	2.464	.626	15.499	1	.000	11.750	3.446	40.064
High school graduate	1.095	.452	5.880	1	.015	2.989	1.234	7.244
Some college/training	.621	.404	2.360	1	.124	1.860	.843	4.105
Race/ethnicity			8.577	4	.073			
Native American	-.411	.839	.240	1	.624	.663	.128	3.434
Other	-.466	.963	.235	1	.628	.627	.095	4.141
Non-Hispanic White	-1.499	.701	4.574	1	.032	.223	.057	.882
Latino, English speaker	-1.344	.638	4.433	1	.035	.261	.075	.911
Prevention orientation	.603	.307	3.849	1	.050	1.827	1.001	3.335
Constant	5.925	1.602	13.681	1	.000	374.2		

R^2 (Nagelkerke) .319
